# Achievements of BSI hospital-wide surveillance in Belgium

**DOI:** 10.1186/1753-6561-5-S6-P234

**Published:** 2011-06-29

**Authors:** I Morales, B Catry

**Affiliations:** 1 Epidemiology, Scientific Institute of Public Health, Brussels, Belgium

## Introduction / objectives

Hospital-wide Blood Stream Infections (BSI) surveillance aims at identifying wards at risk, BSI origin and aetiology to set local and national infection control strategies. To assess its effectiveness and usefulness we studied five years of participation to this voluntary surveillance and the BSI incidence trends.

## Methods

A repeated cross sectional analysis of the national aggregated database was made. The number of participating hospitals and surveillance periods were computed along with the chronological evolution of BSI incidence rates by admissions, patient-days and requested haemocultures. BSI origin and aetiology were also studied.

## Results

Out of 141 acute hospitals, about 80.8 hospitals participated annually with 211.6 surveillance quarters and 5715.8 BSI episodes.

From 2005 to 2009, all indicators fell BSI/1000 admissions from 7.2 to 5.6, BSI/10000 patient-days (9.3 - 7.8), and BSI/1000 haemocultures (2.2 - 1.9). Catheter-related BSI also fell (21.6 - 17.6%), while secondary BSI remained stable (43.5-42.8%) and BSI of unknown origin increased (34.9 - 39.6%). Secondary BSI main source was, by far, urinary tract infections (UTI). Two microorganisms increased over the period: *Escherichia spp.* by 3% and *Streptococcus spp.* by 2%. The intensive care units (ICU) remained the first ward at risk (21.9 to 18.3% BSI were ICU-acquired), but geriatrics rose to a second place (11.6 - 14.0%).

## Conclusion

Although voluntary and hospital-wide, the large participation to this surveillance suggests that practitioners perceive it as useful. The input is large enough to yield valid benchmark for local surveillance and to identify national outliers. Besides overall incidence reduction, the catheter-related BSI fell to an all time low. Future operational priorities include reinforcing ICU and geriatric surveillance, focusing on UTI.

## Disclosure of interest

None declared.

